# 

**Published:** 1874-12

**Authors:** 


### CONTENTS:

PROGRESS IN MEDICAL SCIENCES:
ART. I. Progress of Surgery. By Jno. E. Owens, M.D., Chicago .......... 705
REPORTS OF SOCIETIES:
Chicago Society of Physicians and Surgeons .......... 709
HOSPITALS:
Cook County Hospital. Dr. Powell... 717
CLINICS:
Rush Medical College. Drs. Hay and Gunn .......... 723
SELECTIONS:
Homicide—Suspected Simulation of Insanity .......... 726
Digitalis—Is it a Cardiac Sedative, or Cardiac Stimulant? .......... 734
EDITORS' BOOK TABLE .......... 745
EDITORIAL .......... 758
MORTALITY STATISTICS .......... 760

